# 153. Gone Are the Other Respiratory Viruses During COVID…but the Rhinovirus/Enterovirus “Cockroach” Persists!

**DOI:** 10.1093/ofid/ofab466.153

**Published:** 2021-12-04

**Authors:** Jasjit Singh, Beth Huff, Delma Nieves, Wendi Gornick

**Affiliations:** 1 CHOC Children’s, Orange, CA; 2 UC Irvine / Children’s Hospital Orange County, Orange, California

## Abstract

**Background:**

In a typical winter respiratory season, Influenza A, Influenza B, Respiratory Syncytial Virus (RSV) and human Metapneumovirus (hMPV) infections are common in pediatrics. During the COVID-19 pandemic, we noted a marked decrease in all except for Rhinovirus/Enterovirus at our free-standing quaternary level children’s hospital.

**Methods:**

We prospectively reviewed all patients with positive testing for viral respiratory pathogens from October 1, 2018 through May 29, 2021. Testing was done by polymerase chain reaction (PCR) (BioFire® FilmArray® Respiratory 2 Panel, UT) and by SARS-CoV-2 PCR testing (Cepheid®, CA). The latter may have been done for pre-procedure or admission screening. We submitted 74 specimens to the California Department Public Health (CDPH) for definitive identification and serotyping analysis.

**Results:**

The number of Rhinovirus/Enterovirus (RV/EV) infections was compared with Influenza A & B, RSV, and hMPV over the past 3 years. There was a 152% increase in RV/EV from 2018-2019 to 2020-2021 with near absence of other respiratory viruses (Figure 1). In 2020-2021, RV/EV (N=877, 84%) made up a larger percentage of all viral etiologies compared to 2018-2019 (N=348, 11%) (Figure 2). Healthcare acquired infections (HAI) due to respiratory viruses decreased in 2020-2021 compared to both of the prior seasons, though all cases were due to RV/EV (Figure 3). There were no RV/EV associated deaths. Of 74 submitted, CDPH did typing on 24 samples; all were found to be rhinovirus (RV).

Figure 1. High-Risk Winter Viral Infections 2019-2021.

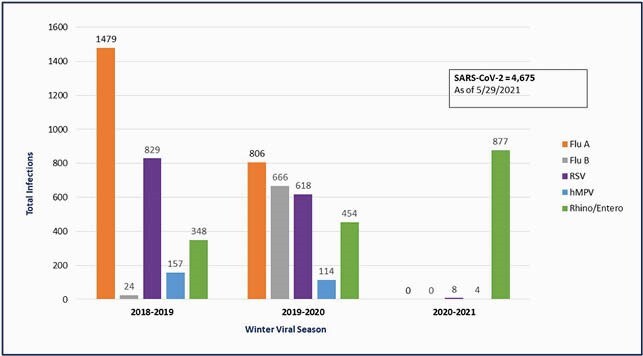

Figure 2. Distribution of Winter Viral Pathogens 2018-2019 Compared to 2020-2021 Season.

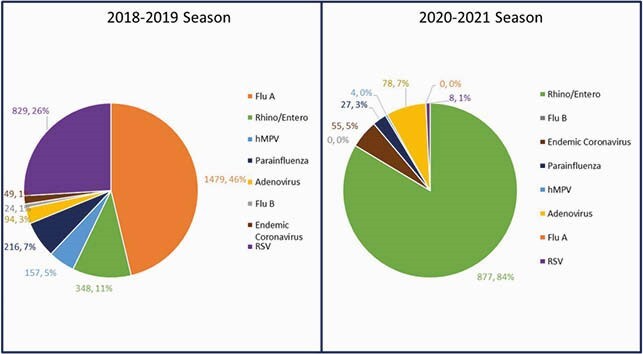

Figure 3. Winter Viral Healthcare Associated Infections 2019-2021.

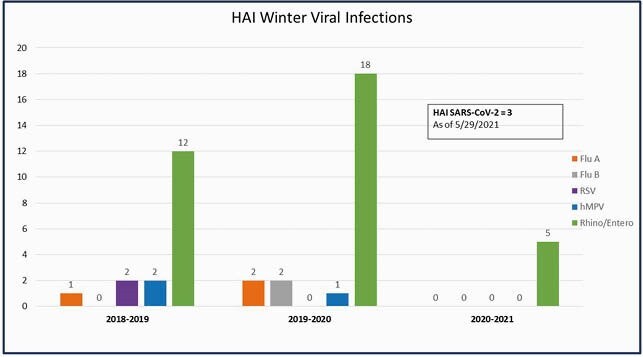

**Conclusion:**

We experienced a marked increase in RV/EV during COVID precautions, despite a near absence of other common respiratory viruses. This was reflected in both our community data and HAI due to respiratory viruses. There was a marked increase in RV/EV starting with week 18 (Figure 4). We hypothesize this is due to schools’ re-opening. Understanding RV epidemiology and transmission is important, as it may inform return to school and work protocols for the upcoming respiratory viral season.

Figure 4. Rhinovirus/Enterovirus by Week for the 2020-2021 Season.

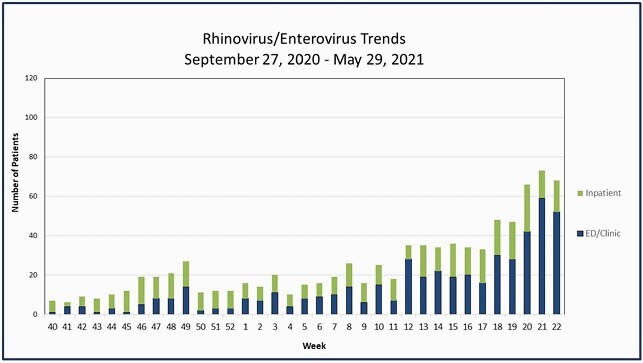

**Disclosures:**

**All Authors**: No reported disclosures

